# Supplementary material for: A Characterization and Functional Analysis of Peroxisome Proliferator-Activated Receptor Gamma Splicing Variants in the Buffalo Mammary Gland
Source: Genes (Basel). 2024 Jun 13;15(6):779. doi: 10.3390/genes15060779 (PMC11203352; doi:10.3390/genes15060779)

**Supplementary Materials:**

**Tables:**

**Table S1** Primer sequences of PPARG variants used in this study

| Genes | Primer sequence（5'→3'） | Access NO. | Length /bp | Application |
|---|---|---|---|---|
| PPARG-X1 | F:ATGCTCTTATTGACCCAGA<br>R:TAAAGTTCGTCCTGGTCGC | XM_044933432.2 | 2771 | CDS clone |
| PPARG-X2 | F:GGGAGAATTCTACTTTCCT<br>R:AGTCTTGGATTCTCCTGCT | XR_006547178.2 | 3743 | CDS clone |
| PPARG-X3 | F:ATGCTCTTATTGACCCAGA<br>R:GGAGCTGTAACATTGAACT | XM_ 044933433.2 | 2724 | CDS clone |
| PPARG-X4 | F:ATGCTCTTATTGACCCAGA<br>R:TAAAGTTCGTCCTGGTCGC | XM_044933434.2 | 2693 | CDS clone |
| PPARG-X5 | F:GTGAAACCCTGGGAGATGC<br>R:TAAAGTTCGTCCTGGTCGC | XM_025271889.3 | 2671 | CDS clone |
| PPARG-X6 | F:ATGGTTGACACAGAGATGC<br>R:TAAAGTTCGTCCTGGTCGC | XM_044933435.2 | 2585 | CDS clone |
| PPARG-X7 | F:ATGGTTGACACAGAGATGC<br>R:TAAAGTTCGTCCTGGTCGC | XM_044933436.2 | 2585 | CDS clone |
| PPARG-X8 | F:ATGGTTGACACAGAGATGC<br>R:TAAAGTTCGTCCTGGTCGC | XM_044933437.2 | 2585 | CDS clone |
| PPARG-X9 | F:ATGGTTGACACAGAGATGC<br>R:TAAAGTTCGTCCTGGTCGC | XM_044933438.2 | 2585 | CDS clone |
| PPARG-X10 | F:ATGGTTGACACAGAGATGC<br>R:TAAAGTTCGTCCTGGTCGC | XM_044933439.2 | 2585 | CDS clone |
| PPARG-X11 | F:ATGGTTGACACAGAGATGC<br>R:TAAAGTTCGTCCTGGTCGC | XM_044933440.2 | 2585 | CDS clone |
| PPARG-X12 | F:ATGGTTGACACAGAGATGC<br>R:TAAAGTTCGTCCTGGTCGC | XM_044933441.2 | 2585 | CDS clone |
| PPARG-X13 | F:ATGCTCTTATTGACCCAGA<br>R:TCAAGGCAGCCAGGGACCT | XM_044933442.2 | 2559 | CDS clone |
| PPARG-X14 | F:ATGCTCTTATTGACCCAGA<br>R:TCAGACGAGGATGCTCAAG | XM_044933443.2 | 1773 | CDS clone |
| PPARG-X15 | F:ATGCTCTTATTGACCCAGA<br>R:TCTGGGTCAATAAGAGCAT | XM_044933444.2 | 1606 | CDS clone |
| PPARG-X16 | F:GTGAAACCCTGGGAGATGC<br>R:AGTCCTTGTAGATTTCCTG | XM_006077448.4 | 1506 | CDS clone |
| PPARG-X17 | F:GCAGTGTTCCGTCCCCCAA<br>R:TAAAGTTCGTCCTGGTCGC | XM_044933445.2 | 1376 | CDS clone |
| PPARG-X18 | F:ATGGCGGAGGCAGTGTTCC<br>R:TAAAGTTCGTCCTGGTCGC | XM_044933446.2 | 1385 | CDS clone |
| PPARG-X19 | F:ATGGCGGAGGCAGTGTTCC<br>R:TAAAGTTCGTCCTGGTCGC | XM_044933447.2 | 1385 | CDS clone |
| PPARG-X20 | F:ATGCTCTTATTGACCCAGA | XM_044933448.2 | 931 | CDS clone |

| | | | | |
|---|---|---|---|---|
| | R:AGCAAACCTGGGCGGTTGA | | | |
| PPARG-X21 | F:AGGTGTGATCTTAACTGTC<br>R:CAAGTCCTTGTAGATTTCC | XM_025271895.2 | 790 | CDS clone |
| PPARG-X17 | F:CCTTGAGCATCCTCGTCT<br>R:TGCCTTCTGATTTGGAAACCAC | XM_044933445.2 | | qRT-qPCR |
| PPARG-X21 | F:GAATTGAAGCTGCCCTTAGGTG<br>R:TCTCCGCTAACAGCTTCTCC | XM_025271895.2 | | qRT-qPCR |
| PPARG-X1.1 | F:AATTCCTCTCCAACCTATC<br>R:TAAGTGAACGCAAGAGCAG | XM_044933432.2 | 1151 | Amplify specific exons |
| PPARG-X1.2 | F:TTTAAAATGCAGCCTCCTC<br>R:TGAGGCCCACGCTGAGAAA | XM_044933432.2 | 191 | Amplify specific exons |
| PPARG-X1.3 | F:CTTCCAAAATTCAGCTGCC<br>R:AATGGAGCTGTAACACTGA | XM_044933432.2 | 814 | Amplify specific exons |
| PPARG-X2.1 | F:CTTCCAAAATTCAGCTGCC<br>R:CACCATGCCCTTTACAACT | XR_006547178.2 | 231 | Amplify specific exons |
| PPARG-X2.2 | F:GAGGTGTTTGGTGAGTGCA<br>R:TCTTCTCTTTGAGTCTTGG | XR_006547178.2 | 99 | Amplify specific exons |
| PPARG-X3 | F:CTGACCACAATTCCTCTCC<br>R:GCAGCAAGTTAAGCATCAA | XM_ 044933433.2 | 1201 | Amplify specific exons |
| PPARG-X5 | F:CAAAACTGACCACAATTCC<br>R:ATGAGACATCCCCACAGCC | XM_025271889.3 | 1041 | Amplify specific exons |
| PPARG-X6 | F:GTGGGTGTGTATCTGTGGT<br>R:ACACGCATCACCAACAGAG | XM_044933435.2 | 248 | Amplify specific exons |
| PPARG-X8 | F:GAAAGTGGGTGTGTATCTG<br>R:ACCTATCTGCGGCTTTTAT | XM_044933437.2 | 100 | Amplify specific exons |
| PPARG-X9 | F:TGTGGGGTCGCGGTAACAG<br>R:CTATCTGCCTCGGTGTTAG | XM_044933438.2 | 378 | Amplify specific exons |
| PPARG-X10 | F:AGGTCTGGCTCCGTCTCCG<br>R:AAGTGCGCCCCAGGCTTAA | XM_044933439.2 | 74 | Amplify specific exons |
| PPARG-X11 | F:GGCCAGAAAACCAAGATAT<br>R:GAGTCTCCGGTCAAGATGT | XM_044933440.2 | 1299 | Amplify specific exons |
| PPARG-X12 | F:CGATGTAAATGCCAAAGTG<br>R:AATGCTCCCAAGTACACAG | XM_044933441.2 | 1560 | Amplify specific exons |
| PPARG-X13.1 | F:TCTCCAACCTATCCGTGTC<br>R:AAAAAAGCTCTGAGTATGC | XM_044933442.2 | 1165 | Amplify specific exons |
| PPARG-X13.2 | F:CGCACTCAGGTTTGTATTT<br>R:CTTGGCTCTGAGTTCACTT | XM_044933442.2 | 173 | Amplify specific exons |
| PPARG-X14 | F:TCTCCAACCTATCCGTGTC<br>R:CTTGGCTCTGAGTTCACTT | XM_044933443.2 | 1175 | Amplify specific exons |
| PPARG-X15 | F:AATGCATTATTCCTCTCAG<br>R:GCTCTGAGTATGCTTTAAG | XM_044933444.2 | 1105 | Amplify specific exons |
| PPARG-X16 | F:TCAGGGAGAATTCTACTTT<br>R:AGGCCACAAGATCAAGTCC | XM_006077448.4 | 990 | Amplify specific exons |
| PPARG-X17 | F:GGAAGCGCAGGAAAGAGGA<br>R:CCTCGAATTGCTACCCGCT | XM_044933445.2 | 271 | Amplify specific exons |
| PPARG-X18 | F:ATTGGGGGCAGGAGGAGAA<br>R:CGGCTCTGGACTGGATTGA | XM_044933446.2 | 421 | Amplify specific exons |

| | | | | |
|---|---|---|---|---|
| PPARG-X19 | F:TGAGTGGTACAGCGCCCTT<br>R:TGAGAGCAGGAAGCGTCAG | XM_044933447.2 | 1590 | Amplify specific exons |
| PPARG-X20 | F:CCACAATTCCTCTCCAACCT<br>R:GCTCTGAGTATGCTTTAAG | XM_044933448.2 | 1171 | Amplify specific exons |
| PPARG-X21 | F:ACTTGCTTCTTTTCTGCCC<br>R:CTGGTGATCTGTGGGAAAA | XM_025271895.2 | 286 | Amplify specific exons |
| ACTB | F:TGGGCATGGAATCCTG<br>R:GGCGCGATGATCTTGAT | NM_001290932.1 | 196 | Amplify specific exons |

Table 2 PPARG-X17 and PPARG-X21 siRNA sequences

| NO. | sequence（5′-3′） |
|---|---|
| siRNA17-5 | Sense: GAAACUCUCUGCCGUGAAUTT<br>Antisense: AUUCACGGCAGAGAGUUUCTT |
| siRNA17-9 | Sense: GAGCCUUUAACGAUCAUGATT<br>Antisense: UCAUGAUCGUUAAAGGCUCTT |
| siRNA17-12 | Sense: GCUGUGCUAUUUGAUUAAATT<br>Antisense: UUUAAUCAAAUAGCACAGCTT |
| siRNA21-5 | Sense: GCGUGCACGAGAUCAUUUATT<br>Antisense: UAAAUGAUCUCGUGCACGCTT |
| siRNA21-6 | Sense: GUUCAACGCACUGGAAUUATT<br>Antisense: UAAUUCCAGUGCGUUGAACTT |
| siRNA21-9 | Sense: GUGCAGCUGUUGCAAGUAATT<br>Antisense: UUACUUGCAACAGCUGCACTT |
| siRNA-NC | Sense: UUCUCCGAACGUGUCACGUTT<br>Antisense: ACGUGACACGUUCGGAGAATT |

Figures:

**Figure S1** Locations of the primers of the PPARG splices

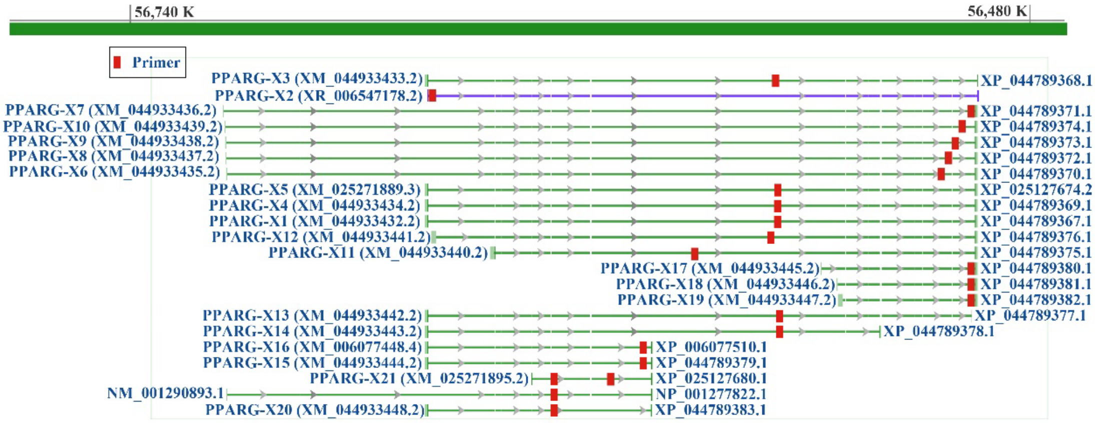


**Figure S2** Clone of PPARG splices from the RNA of buffalo milk somatic cells

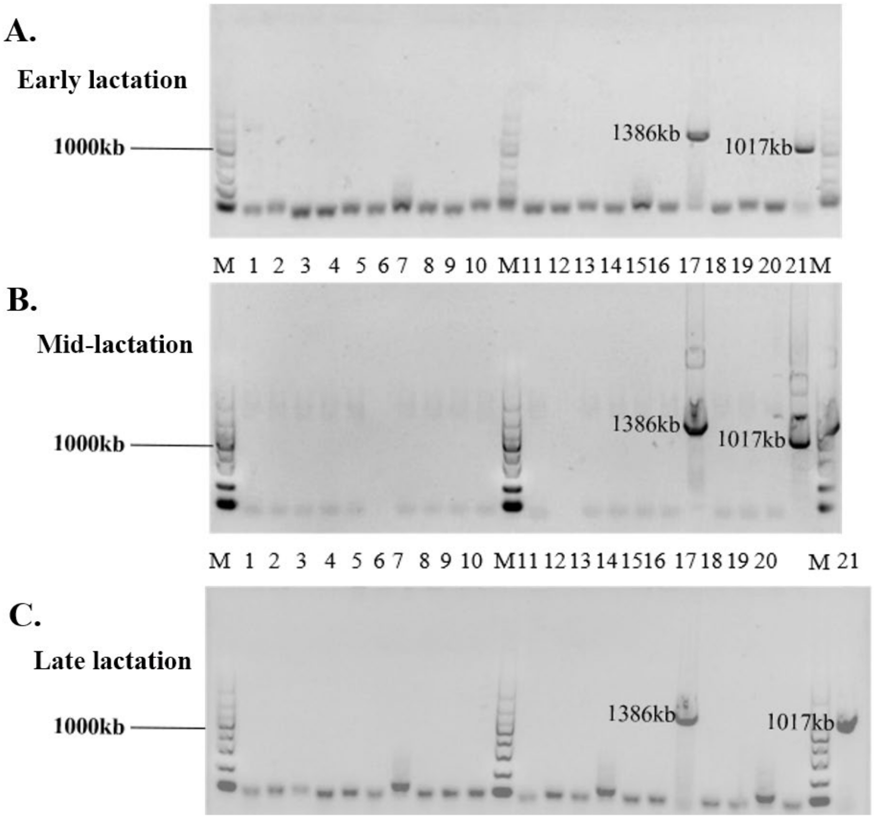

**Figure S3** Blast of the sequences of the cloned PPARG splices

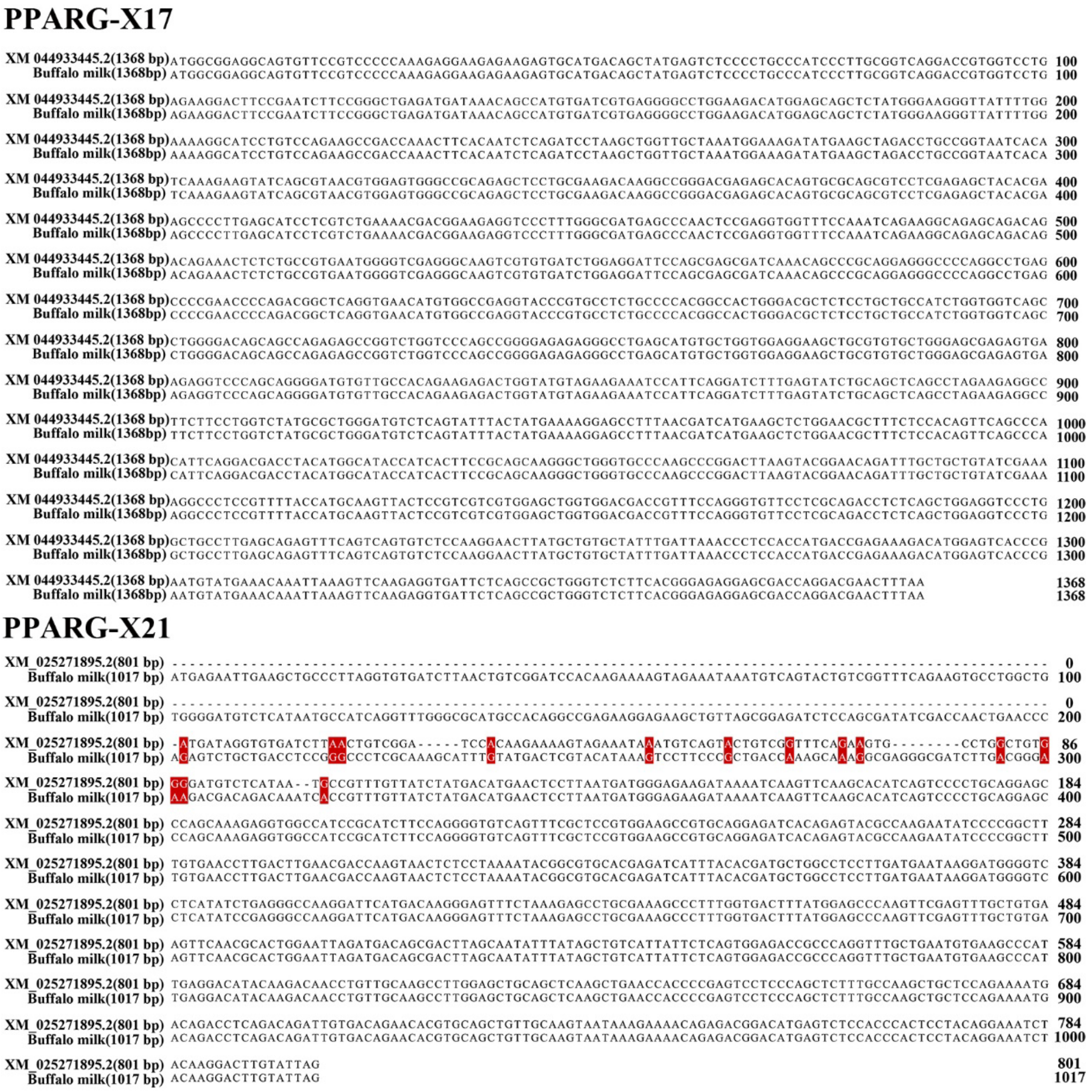


**Figure S4** Comparation of the amino acid sequences of PPARG-X17 and PPARG-X21

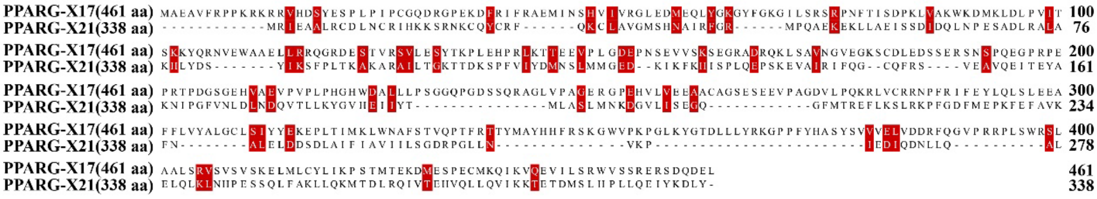

Supplement: Supplementary file 1 [file genes-15-00779-s001.zip › genes-2981702-supplementary.pdf]
